# Henoch-Schönlein purpura in a patient with oesophageal cancer: A case report

**DOI:** 10.1097/MD.0000000000023492

**Published:** 2020-12-04

**Authors:** Haonan Chen, Chao Li, Wenli Ye, Wei Ye, Hui Xu, Qingwei Jiang, Zhen Huo, Xinyan Zhao, Hang Li

**Affiliations:** a Department of Internal Medicine; b Department of Nephrology; c Department of Gastroenterology; d Department of Pathology, Peking Union Medical College Hospital, Chinese Academy of Medical Sciences; e Liver Research Center, Beijing Friendship Hospital, Capital Medical University, Beijing, People's Republic of China.

**Keywords:** Henoch-Schönlein purpura, malignancy, oesophageal cancer, vasculitis

## Abstract

**Rationale:**

Understanding the association between Henoch-Schönlein purpura (HSP) and malignancy is essential for early diagnosis and treatment of the potential lethal disease. To the best of our knowledge, there has been only one published case of HSP coexisting with oesophageal cancer. Here, we report another patient diagnosed with HSP and oesophageal squamous carcinoma simultaneously.

**Patient concerns:**

A 60-year-old Chinese male was referred to our hospital because of intermittent abdominal pain, abdominal distension, melena, lower extremities purpura. Positive laboratory values included pancytopenia, microscopic hematuria, nephrotic proteinuria, hematochezia, hypoalbuminemia, hyperlipidaemia, hypocomplementemia, and increased levels of hepatobiliary enzymes and immunoglobulin (Ig) A. Gastrocolonoscopy showed multiple erosion lesion on descending duodenum, terminal ileum, and ileal flap. Biopsy of these lesions suggested non-specific inflammation.

**Diagnoses:**

HSP (IIIb type) was diagnosed based on renal pathology examination in accordance with the International Study of Kidney Disease in Children (ISKDC) classification. Liver biopsy confirmed the diagnosis of nodular cirrhosis (Ishak 5). Gastroscopy unintentionally revealed three oesophagus lesions. Pathology study suggested intermediate differentiated squamous cell carcinoma (cTNM IB).

**Interventions:**

Before admission, he was administered intravenous Ig 10 g once daily(qd) for 10 days, methylprednisolone 40 mg qd for a week, followed by prednisolone 50 mg qd for almost 8 weeks. Endoscopic submucosal dissection (ESD) was performed to remove all lesions with negative margin after prednisolone was tapered (5 mg per week until 10 mg qd).

**Outcomes:**

Despite prednisone being tapered to 2.5 mg qd within 2 months, complete remission of HSP and esophageal malignancy was achieved after the resection of the esophagus lesions during 12 months follow-up.

**Lessons:**

We report a rare case of oesophageal squamous cell carcinoma initially presented as HSP. This case suggests the importance of evaluating adult patients with HSP for an underlying malignancy.

## 1 Introduction

Henoch-Schönlein purpura is an IgA-immune complex-mediated leukocytoclastic vasculitis manifesting palpable purpura, abdominal pain, arthritis, hematuria, and proteinuria.^[1]^ Neoplasia is a well-documented cause of vasculitis.^[2,3]^ The incidence of vasculitis in patients with malignancy is estimated to be 2.5% to 5%.^[4]^ Although haematological malignancies are 3 to 5 times more common than solid tumors in patients with vasculitis, HSP is notably associated with solid tumors.^[4–7]^ Gastrointestinal tract, lung, and urinary tract are the most commonly affected organs with malignancy in the setting of HSP.^[7]^ Here, we present a rare case with HSP as the initial presentation of squamous cell oesophageal carcinoma.

## 2 Case presentation

A 60-year-old male was referred to our hospital because of intermittent abdominal pain, abdominal distension, melena, lower extremities purpura in July 2018. Blood, urine and faeces routine tests were within normal range in 2014, which remained normal during regular follow up. Until May 2018, he experienced an episode of right upper abdominal pain precipitated with excessive alcohol intake, with darkening urine color, melena, and recurrence purpura in lower extremities. Laboratory results were as follows: Blood routine count showed pancytopenia (white blood cell 2.7 × 10^9^/L, hemoglobin 90–110 g/L, platelet 16 × 10^9^/L). Urinalysis showed hematuria with increased protein excretion (5–7 g/day). Faecal occult blood test was positive for three times. Biochemical examination showed hypoalbuminemia (serum albumin 2.1–3.0 g/dL), hyperlipidaemia (total cholesterol 7–13 mmol/L), with increased level of hepatobiliary enzymes (alanine transaminase 82 U/L, total bilirubin 105.6 umol/L). Baseline creatine level was normal (50–70 μmol/L), but rising substantially after an enhanced computerized tomography examination (150–180 μmol/L). IgA level was increased (5.98 g/L), with decreased level of complement factor 3 (C3 50.5 mg/dL). Erythrocyte sedimentation rate (ESR, 40 mm/h) and C-reactive protein (CRP, 11.89 mg/L) levels were also increased. Ferritin, anti-nuclear antibody, anti-double-stranded DNA antibody, anti-neutrophil cytoplasmic antibody, anti-glomerular basement membrane antibody, serum, and urine protein electrophoresis, cryoglobulin, anti-Hepatitis B surface antigen, anti-hepatitis C virus antibody were all within normal range or negative. Enhanced abdominal CT showed duodenum, part of intestine, and whole colon were thickened with enhancement. Large amount of ascites were detected. Positron emission tomography yielded no obvious sign of solid tumor. Gastroscopy showed multiple erosion lesion on descending duodenum. Colonoscopy showed diffused oedema with patchy ulceration in terminal ileum and ileal flap. Biopsy of these lesions suggested non-specific inflammation. Further investigation of ascites revealed no sign of infection or malignancy. Before he was referred to our hospital, he was administered intravenous Ig 10 g qd for 10 days, methylprednisolone 40 mg qd for a week, followed by prednisolone 50 mg qd for almost 8 weeks. He also took furosemide 40 mg qd and spironolactone 100 mg qd for ascites. He had a history of well-controlled hypertension for 7 years. He suffered an episode of acute myocardial infarction in 2012, receiving three stents and dual antiplatelet treatment (aspirin and clopidogrel) until the presence of melena in May 2018. He had been drinking alcohol 40 g/day for 10 years and smoking 10 cigarettes per day for 40 years. The patient's father had oesophagus cancer and his mother had lymphoma. On admission, his vital signs were as follows: body temperature 37.6°C, blood pressure 145/86 mm Hg, pulse rate 58 beats/min, regular, and SpO2 96% (room air). Physical examination showed liver palms on both hands and one spider angioma on his chest. Cardiac, pulmonary, and abdominal examinations were negative. No obvious edema or purpura was seen in lower extremities.

**Figure 1:**
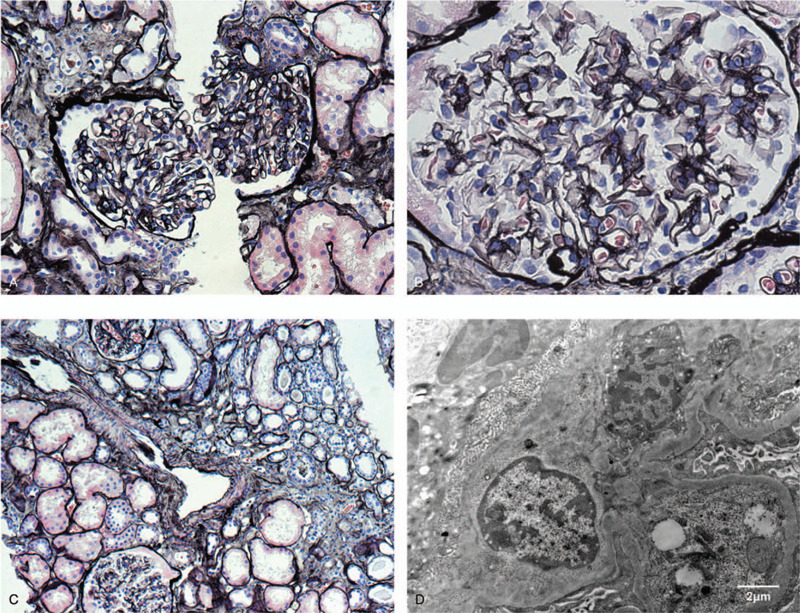
Renal biopsy findings of the case. (A) The left glomerulus was normal while the right one showed mild mesangial hypercellularity (Jones silver stain, ×200). (B) The glomerulus showed mild segmental mesangial hypercellularity without crescent or necrotizing lesion (Jones silver stain, ×400). (C) Light micrograph showed focal interstitial inflammation, fibrosis and tubular atrophy (Jones silver stain, ×100). (D) Electron micrograph demonstrated the dense deposits in mesangial area (×8000).

Laboratory tests in our center showed a normal whole blood count, negative faecal occult blood test. Urinalysis revealed microscopic haematuria and proteinuria (24 h urine protein 2.41 g). Creatine level was continuously fallen (117→108 μmol/L). Renal biopsy showed 15 glomeruli; 1 segmental sclerosis and 1 cellular crescent with diffused segmental mesangial cell proliferation and increased mesangial matrix. No obvious thickening was observed in glomerular basement membrane (GBM). Immunofluorescence staining was positive for IgA in mesangial with focal granule-like deposition (Fig. 1). Thus, a diagnosis of Henoch-Schönlein purpura (IIIb type) was established. Liver function test revealed a low serum albumin level (24–29 g/L), with normal range of ALT and TBIL. Transient elastography revealed a high liver stiffness value (24.1 kPa). Liver biopsy showed nodular cirrhosis (Ishak 5). Gastroscopy showed multiple scar-like changes on duodenum, suggesting good steroid response. Surprisingly, three active lesions were found at lower esophagus, with irregular shape and obvious hyperaemia. Further endoscopy examination included endoscopic ultrasonography (early esophagus cancer, probably SM1) and magnifying gastroscopy (IPCL b1-b2 with NBI observation). Pathological examination confirmed a diagnosis of intermediate differentiated oesophageal squamous carcinoma.

We followed up with prednisone 50 mg qd for another 2 weeks without other immunopressive therapy. After the esophageal malignancy was diagnosed, we tapered prednisone 5 mg/week until 10 mg qd, for the potential risk of high dose steroid regimen perioperatively. In September and November 2018, the patient received two ESD procedures with complete resection of three esophagus lesions. Despite prednisone being tapered to 2.5 mg qd within 2 months, 24 h urine protein remained minimal (6.12→0.13 g). Complete remission of HSP and esophageal malignancy was achieved after the resection of the esophagus lesions during 12 months follow-up.

## 3 Discussion

HSP is a systemic vasculitis involving small blood vessels, most notably the skin, gastrointestinal tract, and glomeruli, accompanied by arthralgia or arthritis.^[8]^ While the exact cause of HSP remains unknown,^[7,9]^ several studies have reported malignancy as a rare cause of HSP. Pertuiset et al retrospectively reviewed 14 adults diagnosed with HSP and malignancy was found in 4 patients with HSP and malignancy were older and more likely to be male.^[5]^ Mitsui et al reported 23 patients (all aged ≥41 years) diagnosed with HSP had underlying malignancies. 88.9% tumors originated from solid organs. One patient was diagnosed with early sigmoid colon cancer during the treatment of HSP. After resection of the tumor, HSP was simultaneously cured and no recurrence was observed.^[6]^ Podjasek et al reviewed a total of 47 cases with solid malignancy-associated HSP. Patients were predominantly male (70%) with a mean age of 62 years. Lung, urinary system, and gastric were the most frequently affected organs.^[7]^ In terms of esophagus cancer, Mita et al reported a 69-year-old man suffered from paraneoplastic vasculitis associated with oesophageal and gastric carcinoma in 1999.^[10]^ Noticeably, Weiler-Bisig et al reported the first case of HSP associated with oesophagus cancer in 2005.^[11]^

Fortin PR categorized the relationship between vasculitis and malignancy into three broad clinical groups: vasculitide-associated malignancies or true paraneoplastic syndrome (two conditions starting at the same time, following a parallel clinical course), malignancies masquerading as vasculitides (such as angiocentric or intravascular neoplasms), and vasculitides masquerading as malignancies (such as granulomatosis with polyangiitis).^[3]^ Several hypotheses have been proposed concerning the association between malignancy and vasculitis^[2,3,6,7,12,13]^:

1. Abnormal immune complex deposited within vascular walls caused by overproduction of tumor associated antigens and antibodies.
2. Excessively generated immune complex that cannot be appropriately cleared.
3. Common antigens presented both at the malignant and endothelial cell, inducing immune injury not only to tumor cells but also to normal endothelium.
4. Aberrant inflammatory cytokines produced either by malignant tumor cells or through tumor microenvironments, causing endothelial damage and increased vascular permeability, inflammation, and fibrosis.
5. Hyperviscous state caused by malignancy that can lead to endothelial damage and increase the contact time for the deposition of immune complexes.

Concerning the pathogenesis of HSP, endothelial damage, perivascular leukocytic infiltrates, chemokines, and cytokines are deemed important factors.^[14]^ Vascular deposition of IgA1-containing immune complexes also plays an essential role.^[15–17]^ Beauflls et al studied the association between renal pathology and solid tumor in 129 cadavers. Immune complex deposits were observed in 22 of the neoplastic patients. These deposits were usually located in mesangial area. IgA deposits were present in 8 of 22 patients.^[2]^

In conclusion, we presented a patient with intermittent abdominal pain, abdominal distension, melena, and lower extremities purpura. The patient was later diagnosed with oesophageal squamous carcinoma but initially presented as HSP and cirrhosis. Our patient showed an increased level of multiple inflammentary factors, suggesting intricate interaction between upregulated autoimmune state and malignancy. Since the efficiency of glucocorticoid for renal injury associated with HSP has not been fully established,^[9]^ we assume the rapid remission of such severe renal damage was, at least in part related to the resection of oesophageal malignancy. Noticeably, this patient also had mild cirrhosis which may lead to lower clearance rate of immune complexes. Therefore, based on previous literature and our case, we recommend that adults, especially older male (around 60 years old) who present with unexplained HSP, be evaluated for an underlying malignancy.

## Acknowledgments

Renal pathology slide preparing by Lin Duan, Yan Li, Xiwei Yan were greatly appreciated.

## Author contributions

CHN and LC wrote the manuscript and was the treating physician for the patient. LH analyzed the clinical course and helped draft the manuscript. YW and YWL reviewed the renal pathology independently. XH and JQW performed the oesophageal lesion biopsy and ESD. HZ reviewed the oesophageal pathology. ZXY reviewed the liver pathology. All authors read and approved the final manuscript.

**Investigation:** Wenli Ye, Wei Ye, Hui Xu, Qingwei Jiang, Zhen Huo, Xinyan Zhao.

**Supervision:** Chao Li, Hang Li.

**Writing – original draft:** Haonan Chen.

**Writing – review & editing:** Haonan Chen, Chao Li, Hang Li.

## References

[1] Gaskill N Guido B Magro CM Recurrent adult onset Henoch-Schonlein Purpura: a case report. Dermatol Online J 2016;22:27617937

[2] Beaufils H Jouanneau C Chomette G Kidney and cancer: results of immunofluorescence microscopy. Nephron 1985;40:303–8. 4010844 10.1159/000183483

[3] Fortin PR Vasculitides associated with malignancy. Curr Opin Rheumatol 1996;8:30–3. 8867536 10.1097/00002281-199601000-00005

[4] Zurada JM Ward KM Grossman ME Henoch-Schonlein purpura associated with malignancy in adults. J Am Acad Dermatol 2006;55: 5 Suppl: S65–70. 17052537 10.1016/j.jaad.2005.10.011

[5] Pertuiset E Liote F Launay-Russ E Adult Henoch-Schonlein purpura associated with malignancy. Semin Arthritis Rheum 2000;29:360–7. 10924021 10.1053/sarh.2000.6988

[6] Mitsui H Shibagaki N Kawamura T A clinical study of Henoch-Schonlein Purpura associated with malignancy. J Eur Acad Dermatol Venereol 2009;23:394–401. 19207675 10.1111/j.1468-3083.2008.03065.x

[7] Podjasek JO Wetter DA Pittelkow MR Henoch-Schonlein purpura associated with solid-organ malignancies: three case reports and a literature review. Acta Derm Venereol 2012;92:388–92. 22293661 10.2340/00015555-1288

[8] Jennette JC Falk RJ Small-vessel vasculitis. N Engl J Med 1997;337:1512–23. 9366584 10.1056/NEJM199711203372106

[9] Hetland LE Susrud KS Lindahl KH Henoch-Schonlein Purpura: a literature review. Acta Derm Venereol 2017;97:1160–6. 28654132 10.2340/00015555-2733

[10] Mita T Nakanishi Y Ochiai A Paraneoplastic vasculitis associated with esophageal carcinoma. Pathol Int 1999;49:643–7. 10504526 10.1046/j.1440-1827.1999.00925.x

[11] Weiler-Bisig D Ettlin G Brink T Henoch-schonlein purpura associated with esophagus carcinoma and adenocarcinoma of the lung. Clin Nephrol 2005;63:302–4. 15847258 10.5414/cnp63302

[12] Chen T Guo ZP Li MM Tumour necrosis factor-like weak inducer of apoptosis (TWEAK), an important mediator of endothelial inflammation, is associated with the pathogenesis of Henoch-Schonlein purpura. Clin Exp Immunol 2011;166:64–71. 21762126 10.1111/j.1365-2249.2011.04442.x PMC3193920

[13] Magro CM Crowson AN A clinical and histologic study of 37 cases of immunoglobulin A-associated vasculitis. Am J Dermatopathol 1999;21:234–40. 10380044 10.1097/00000372-199906000-00005

[14] Dursun I Dusunsel R Poyrazoglu HM Circulating endothelial microparticles in children with Henoch-Schonlein purpura; preliminary results. Rheumatol Int 2011;31:1595–600. 20499069 10.1007/s00296-010-1528-9

[15] Sohagia AB Gunturu SG Tong TR Henoch-schonlein purpura-a case report and review of the literature. Gastroenterol Res Pract 2010;2010:597648. 20508739 10.1155/2010/597648 PMC2874920

[16] Egan CA Taylor TB Meyer LJ IgA1 is the major IgA subclass in cutaneous blood vessels in Henoch-Schonlein purpura. Brit J Dermatol 1999;141:859–62. 10583167 10.1046/j.1365-2133.1999.03159.x

[17] Davin JC Ten Berge IJ Weening JJ What is the difference between IgA nephropathy and Henoch-Schonlein purpura nephritis? Kidney Int 2001;59:823–34. 11231337 10.1046/j.1523-1755.2001.059003823.x

